# Impact of Protease Inhibitor-Based Highly Active Antiretroviral Therapy on Fetal Subcutaneous Fat Tissue in HIV-Pregnant Women in a Middle-Income Country

**DOI:** 10.3390/v16010010

**Published:** 2023-12-20

**Authors:** Hector Borboa-Olivares, Guadalupe Estrada-Gutierrez, Raigam Jafet Martinez-Portilla, Salvador Espino-y-Sosa, Arturo Flores-Pliego, Aurora Espejel-Nuñez, Ignacio Camacho-Arroyo, Juan Mario Solis-Paredes, Jose Rafael Villafan-Bernal, Johnatan Torres-Torres

**Affiliations:** 1 Community Interventions Research Branch, Instituto Nacional de Perinatología, Mexico City 11000, Mexico; 2 Research Direction, Instituto Nacional de Perinatologia, Mexico City 11000, Mexico; guadalupe.estrada@inper.gob.mx; 3 Clinical Research Branch, Instituto Nacional de Perinatología Isidro Espinosa de los Reyes, Mexico City 11000, Mexicosalvadorespino@gmail.com (S.E.-y.-S.); juan.mario.sp@gmail.com (J.M.S.-P.); 4 Department of Immunobiochemistry, Instituto Nacional de Perinatología, Mexico City 11000, Mexico; arturo.flores@inper.gob.mx (A.F.-P.); aurora.espejel@inper.gob.mx (A.E.-N.); 5 Unidad de Investigación en Reproducción Humana, Instituto Nacional de Perinatología-Facultad de Química, Universidad Nacional Autónoma de México, Mexico City 11000, Mexico; camachoarroyo@gmail.com; 6 Laboratory of Immunogenomics and Metabolic Diseases, Instituto Nacional de Medicina Genomica, Mexico City 14610, Mexico; joravibe@gmail.com

**Keywords:** HIV-pregnant women, protease inhibitor-based highly active antiretroviral therapy, subcutaneous fat tissue, neonatal glycemia, fetal fat redistribution

## Abstract

Background: HIV infection continues to be a global public health challenge, affecting approximately 1.7 million reproductive-aged women. Protease inhibitor-based highly active antiretroviral therapy (PI-HAART) has significantly reduced the risk of vertical transmission of HIV from mother to child. Nevertheless, concerns linger regarding the long-term effects, particularly on body composition, notably subcutaneous fat tissue (SFT). Although HIV-associated lipodystrophy syndrome (LS) has been well documented in adults and older children, its impact on fetuses exposed to PI-HAART remains underexplored. This study aims to evaluate SFT in the fetuses of HIV-pregnant women exposed to PI-HAART, assessing the potential clinical implications. Methods: We conducted a comparative study between HIV-pregnant women receiving PI-HAART and an HIV-negative control group. Fetometry measurements were obtained via 3D ultrasound. SFT in the fetal arm and thigh segments was assessed. Data were analyzed using lineal multivariate regression and receiver-operating characteristics (ROC)-curve analysis. Results: Fetuses exposed to PI-HAART exhibited a significant reduction in subcutaneous fat, particularly in the proximal third-middle union of the femur (coefficient: −2.588, *p* = 0.042). This reduction was correlated with lower newborn serum glucose levels (65.7 vs. 56.1, *p* = 0.007; coefficient: −1.277, *p* = 0.045). Conclusions: Our study sheds light on the connection between PI-HAART, fetal subcutaneous fat, and neonatal health. These findings might reveal the long-lasting effects of PI-HAART on newborns and children’s well-being. Our results emphasize the need for a more balanced approach to managing pregnant women with HIV in developing countries and open new venues for research on the impact of intrauterine PI-HAART exposure on energy metabolism and fetal programming.

## 1. Introduction

HIV infection remains a global public health challenge, with approximately 1.7 million women of reproductive age living with the virus [1,2]. The use of protease inhibitor-based highly active antiretroviral therapy (PI-HAART) during pregnancy has played a pivotal role in reducing the risk of vertical transmission of HIV from mother to child [3,4,5]. PI-HAART, however, is not without its complexities, and its long-term effects on maternal and fetal health are subjects of growing interest and concern. One notable area of concern is the potential impact of PI-HAART on body composition, specifically the subcutaneous fatty tissue (SFT) [6,7]. Alterations in body fat distribution have been observed in individuals living with HIV, commonly referred to as HIV-associated lipodystrophy syndrome (LS) [8,9]. LS manifests in approximately 80% of individuals undergoing PI-HAART and is considered an antiretroviral treatment side effect [10,11]. These alterations, characterized by the loss of fatty tissue in certain areas and redistribution in others, have been extensively studied in adults and older children receiving antiretroviral treatment [8]. However, limited research has been conducted to investigate these effects in fetuses exposed to PI-HAART in utero [12,13].

This study seeks to address this knowledge gap by evaluating the SFT area in the arms and legs of the fetuses of HIV-pregnant women receiving PI-HAART. We hypothesize that PI-HAART exposure during pregnancy may lead to alterations in fetal SFT distribution, similar to those observed in older age groups, and that these alterations may have clinical implications for neonatal and childhood health. 

In Mexico and Latin America, the PI-HAART regimen used may sometimes be different from that used elsewhere in the world [14]. While the PI-HAART regimen used in our study may not be the same as that employed globally, it is representative of what is prescribed in developing countries.

The efficacy of PI-HAART in reducing vertical transmission of HIV has been widely demonstrated in middle-income countries, leading to its implementation in many of these nations, including Mexico [15]. However, the reality is that, despite its efficacy in preventing HIV transmission, it is also essential to consider its potential long-term consequences for maternal and fetal health.

Therefore, the aim of our study was to evaluate the SFT area in the arms and legs of fetuses exposed and not exposed to PI-HAART in HIV-positive and negative pregnant women.

## 2. Materials and Methods

### 2.1. Study Design and Participants

This study was designed as a comparative study between two groups of pregnant women: a group of women seropositive for the human immunodeficiency virus (HIV) who received PI-HAART during pregnancy and a control group composed of women without HIV infection.

Participant selection was conducted at a reference prenatal care center between March 2016 and July 2017. Pregnant women within a gestational age range of 36.0 to 36.6 weeks were included. Thirty-four singleton pregnant women were conveniently selected during their third-trimester ultrasound appointments.

Study Group (HIV-pregnant women): We enrolled seventeen women who were seropositive for HIV and demonstrated consistent adherence to antiretroviral treatment before and during pregnancy. All participants in this group maintained a stage I classification, according to the CDC (Centers for Disease Control and Prevention), throughout their pregnancy. Stage I signifies confirmed HIV infection, with an undetectable viral load and a CD4 concentration exceeding 499/μL. This stringent criterion was applied to minimize the possibility of our findings being influenced by the HIV infection. All participants initiated antiretroviral treatment before pregnancy and continued with the same drug regimen of Lopinavir 400 mg/Ritonavir 100 mg, per day, divided into two doses + Lamivudine 300 mg/Zidovudine 600 mg, per day, divided into two doses [16]. This treatment scheme was recommended for pregnant patients by the International Antiviral Society-USA Panel at the time of recruitment. Currently, the guidelines have suggested changing the use of lopinavir/ritonavir for other new-generation antiretrovirals (darunavir/cobicistat–elvitegravir/cobicistat–zidovudine/lamivudine) [13] with better efficacy in reducing viral load and improving CD4 lymphocyte count; however, the effects reported about lipodystrophy syndrome and other side effects of the drugs remain the same. In developing countries, sometimes these new drugs are not available, so the use of lopinavir/ritonavir is still suggested for pregnant patients [13,17].

Control Group: We recruited seventeen women who tested seronegative for HIV, carefully matching them with the study group based on gestational age, fetal gender, maternal BMI, and weight gain at the time of the study. None of the participants in the control group had comorbidities that could affect fetal growth or body composition. All participants underwent a C-section delivery, determined by obstetric considerations.

Women were recruited between 36 and 36.6 weeks of gestation, as confirmed by the last menstrual period and first-trimester ultrasound. All recruited women received routine prenatal care at INPer, and relevant clinical data were extracted from their medical records. The Department of Infectious Diseases, in collaboration with the Department of Obstetrics, oversaw the medical follow-up of the HIV-positive patients, determining the timing of C-section delivery and implementing prophylactic measures to prevent vertical transmission of the disease. C-section deliveries were performed in accordance with institutional and some international recommendations. Participants were monitored until the completion of their pregnancies, allowing for the collection of perinatal outcomes. While no sample size calculation was conducted in advance, post hoc power analysis confirmed that the statistical power exceeded 80% for all variables exhibiting significant differences.

### 2.2. Ultrasound Fetometry and Fat Mass Area Assessment 

Fetometry measurements were conducted using a Voluson E8 (GE Healthcare, Chicago, IL, USA)^®^ 3D ultrasound equipped with a volumetric transducer (4–8 MHz). Key fetal metrics, such as biparietal diameter, head circumference, abdominal circumference, and femoral length, were taken to estimate fetal weight, employing the Hadlock 2 formula. Fetal weight percentiles were calculated based on gestational age, utilizing the Hadlock reference values preloaded in the ultrasound system.

Volumetry assessments were carried out on the fetal arm and thigh (humerus/femur), positioned anterior to the maternal abdominal wall. The transducer was placed as close to the limb as possible without applying pressure, ensuring no interference from fetal or maternal movements. The volumetric transducer, operating at a frequency of 4–8 MHz, utilized the initial settings employed in the 2D evaluation, with adjustments made to contrast and zoom to encompass 70–80% of the screen area. The focus was set on the region of interest, and gains were optimized to enhance image quality. A volume acquisition angle of 80° was chosen, ensuring accurate centering of the limb (Supplementary Figure S1).

The quality of the images was influenced by the exposure speed, and a rotational scan was employed in a sagittal Z plane with an acquisition time of 10 s. Subsequently, in the offline assessment phase, the ViewPoint program from GE Healthcare was utilized. The “explore submenu” was selected, and the acquired file was loaded. The sagittal plane of the bone was displayed as the main screen, with the proximal epiphysis lateralized to the left. A Sepia filter was applied to accentuate the boundaries of lean and fat mass. The TUI (Tomographic Ultrasound Imaging) tool was used to define three tomographic slices, each with the diaphysis centered, including the central position and one to the right and one to the left.

The fat mass area was calculated by subtracting the central area representing lean mass, composed of bone and muscle, from the total area captured in the image. A minimum of two measurements were taken for each tomographic plane, and the measurement with the highest quality was selected for analysis. Three different planes of the humerus/femur were utilized: the union of the proximal third with the middle third, the middle of the bone, and the union of the distal third with the middle third. The image acquisition and subsequent offline analysis were performed by three ultrasound specialists with expertise in maternal–fetal medicine, all adhering to a standardized technique. Prior to the study, the technique was harmonized among these three operators, and the inter- and intra-observer variability was assessed, yielding an intraclass correlation coefficient exceeding 0.90 for all three selected planes.

### 2.3. Outcomes

The primary outcome of this study was to assess the SFT area in the arms and legs of fetuses exposed to PI-HAART during pregnancy in HIV-positive women. The secondary outcomes included the assessment of potential clinical implications stemming from any observed alterations in fetal subcutaneous fat, which include birth weight, abdominal circumference, and serum glucose.

### 2.4. Statistical Analysis

Descriptive and inferential statistics were used. The distribution of the data was assessed using the Kolmogorov–Smirnov test. Quantitative variables were reported as median and interquartile range, while qualitative data were reported as *n* (%). Differences in variables between HIV-positive and control group cases were evaluated using the Mann–Whitney *U*-test or chi-square test.

Lineal regression analysis was performed to assess the association between PI-HAART and the primary and secondary outcomes. Following linear regression, the performance of the model was evaluated by receiver-operating characteristic (ROC)-curve analysis. Statistical analysis was performed using Stata 17 (StataCorp., College Station, TX, USA). 

## 3. Results

### 3.1. Description and Characteristics of the Study Groups

The demographic characteristics of the included population are presented in Supplementary Table S1. Among the enrolled pregnant women, a majority were married (10/34, 29.4%). The median age of the participants was 31 years, with only 14.7% being employed. Notably, the majority of pregnant women fell within the lower-middle-class category, accounting for 64.7% of the total population. Baseline characteristics were similar between the study groups (Table 1). The HIV-positive group exhibits lower serum glucose concentrations in newborns compared to the control group (65.7 vs. 56.1, *p* = 0.007). All participants initiated antiretroviral treatment before pregnancy and continued with the same drug regimen: Lopinavir 400 mg/Ritonavir 100 mg, per day, divided into two doses + Lamivudine 300 mg/Zidovudine 600 mg, per day, divided into two doses. The mean time from the start of treatment for the included patients to the time of recruitment was 26.79 months (SD 11.96 months). All neonates born from mothers with HIV had a negative result for virus detection at birth.

### 3.2. Association between PI-HAART and Fetal SFT

**Figure 1:**
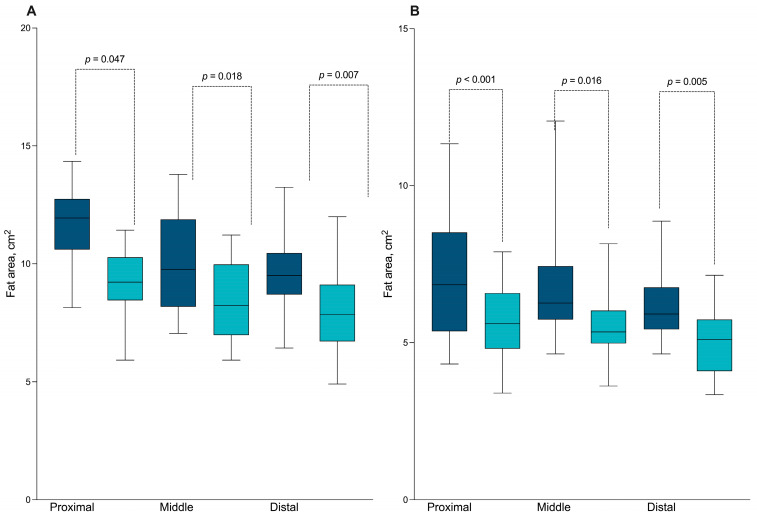
Differences in the fat area around the fetal humerus (**A**,**B**) femur were analyzed with 4D-view tomographic ultrasound imaging between the control group (**−**) and the HIV-positive group (**−**).

We evaluated the subcutaneous fatty tissue in the arms and legs of fetuses exposed to PI-HAART in HIV-positive pregnant women and compared these findings with a control group (Table 2). Figure 1 illustrates that a significantly smaller fat area was observed in the three selected humerus and femur segments of fetuses from women who were HIV-positive compared to the control group. These segments included the junction of the proximal third and middle third (*p* = 0.047), the middle third (*p* = 0.018), and the junction of the distal third and middle third (*p* = 0.007). In the femur, a decrease in fat area was also detected at the junction of the proximal third and middle third (*p* < 0.001), the middle third (*p* = 0.016), and the junction of the distal third and middle third (*p* = 0.005).

In the multivariate linear regression analysis, we identified a significant association between PI-HAART and a reduction in fetal subcutaneous fat tissue (SFT), specifically in the proximal third-middle union of the femur (coefficient: −2.588, *p* = 0.042). Additionally, we observed lower levels of newborn serum glucose associated with PI-HAART exposure (coefficient: −1.277, *p* = 0.045) (Table 3).

### 3.3. Association between Fetal SFT and Serum Glucose in the Newborn

**Figure 2:**
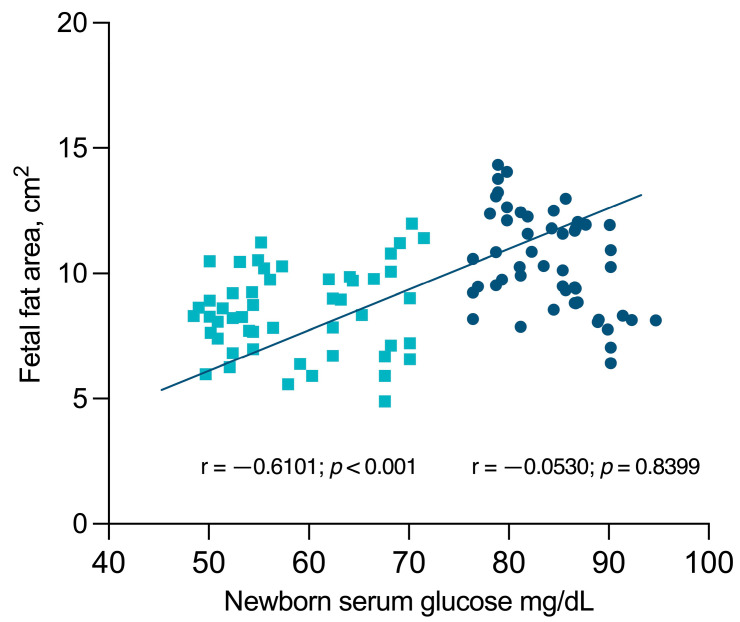
Distribution of newborn serum glucose and fetal subcutaneous fat tissue between the HIV-positive group (**−**) and the control group (**−**).

The distribution of newborn serum glucose and fetal subcutaneous fat tissue differed significantly between the two groups (Figure 2), with the HIV group exhibiting lower fetal subcutaneous fat, consequently resulting in lower serum glucose levels in the newborns. The Spearman correlation analysis unveiled significant correlations between SFT and neonatal glycemia in HIV-pregnant women (r = −0.6101; *p* < 0.001) but not in the control group (r = −0.0530; *p* = 0.8399). The final multivariate model incorporated fetal subcutaneous fat tissue located in the proximal third to middle union of the femur, establishing it as an independent indicator of reduced glucose levels in newborns. Through this analysis, a significant correlation emerged, highlighting the association between fetal subcutaneous fat in the specified proximal third to middle union of the femur, treated as a continuous variable, and the observed decrease in serum glucose levels in newborns (OR 0.391; 95%CI 0.200–0.761, *p* = 0.006).

**Figure 3:**
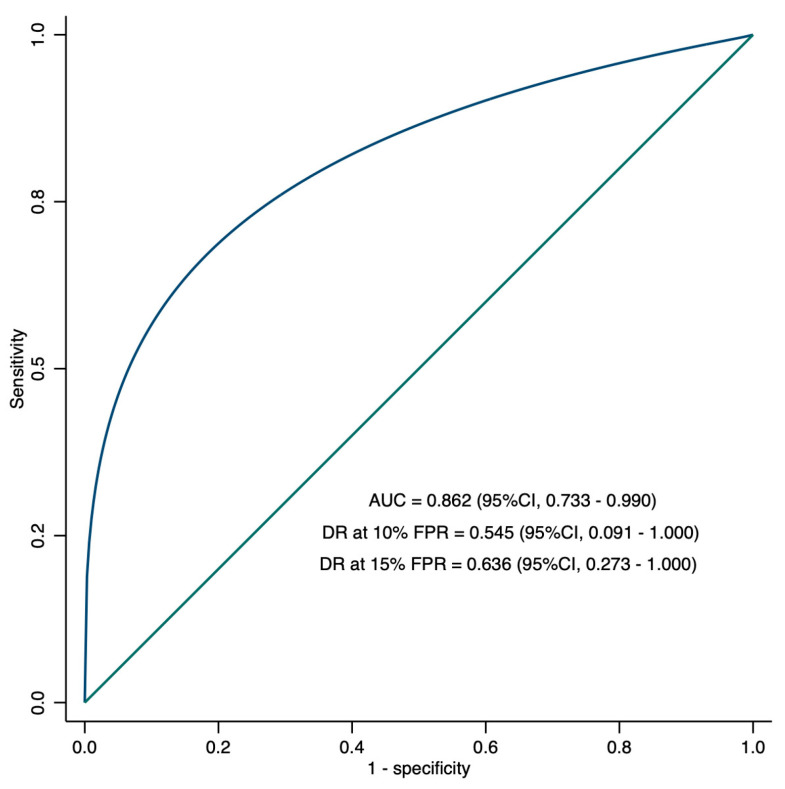
Receiver-operating-characteristics (ROC) curves showing the performance of maternal BMI and fetal subcutaneous fat in the proximal third-middle union of the femur in the prediction of decreased newborn serum glucose (area under the ROC curve (AUC), detection rate (DR), and confidence interval (CI)).

We evaluated the predictive ability of fetal subcutaneous fat in the proximal third-middle union of the femur for decreased newborn serum glucose using a 60 mg/dL cut-off. The resulting area under the ROC curve (AUC) was 0.862 (95%CI, 0.733–0.990), which indicates a 63.6% detection rate for decreased newborn serum glucose at a 15% false positive rate (Figure 3).

## 4. Discussion

### 4.1. Main Findings

Our results demonstrate a significant reduction of SFT in the proximal and middle third of the femur in those fetuses exposed to PI-HAART during pregnancy. This reduction in fetal fat is associated with lower serum glucose levels at birth in the newborns of HIV-positive mothers. Although the specific PI-HAART regimen used in our study may differ from what is used in other countries, our discoveries highlight the relationship between PI-HAART, fetal subcutaneous fat, and neonatal metabolic outcomes, emphasizing the importance of monitoring the changes in fat deposits and neonatal glycemia induced by PI-HAART to increase our knowledge on this topic and prevent perinatal complications related to PI-HAART.

### 4.2. Comparison with Existing Literature

Our findings align with previous research on PI-HAART and body composition alterations in individuals living with HIV, commonly referred to as HIV-associated lipodystrophy syndrome. These alterations are characterized by the loss of fatty tissue in certain areas and its redistribution in others and have been well-documented in adults and older children receiving antiretroviral treatment [8,9,18]. These findings have raised concerns about metabolic and cardiovascular health in individuals living with HIV [19,20,21]. However, limited research has explored these effects in fetuses exposed to PI-HAART in utero. Our study bridges this knowledge gap and contributes to the existing literature by demonstrating that fetal subcutaneous fat is indeed influenced by maternal PI-HAART exposure during pregnancy. 

Although we stopped short of definitively associating lipodystrophy syndrome with these fetuses, it is clear that at least one of its components is evident in our findings. It is important to note that diagnosing dyslipidemia and insulin resistance in a newborn remains a topic of debate due to the absence of a consensus. Nonetheless, it is reasonable to infer that the observed results are linked to PI-HAART exposure, especially when considering the meticulous control of other variables potentially affecting fetal body composition in our patient selection process, which showed no significant disparities in our conducted analyses.

Significantly, this study presents a novel contribution to the current body of literature by unveiling a direct connection between maternal PI-HAART exposure and fetal fat distribution. To the best of our knowledge, this investigation stands as the first to demonstrate a reduction in the subcutaneous fatty tissue area in the extremities of fetuses born to HIV-positive mothers who received antiretroviral treatment. Thus, this study demonstrates fat redistribution in fetuses from HIV-positive mothers, resulting in abnormalities in glucose metabolism. Such findings suggest a potential impact of antiretrovirals during pregnancy on biological networks involved in fat distribution and glucose metabolism that should be studied and characterized in depth in the future through all available genomic, proteomic, lipidomic, and metabolic approaches. 

However, it is essential to acknowledge that not all antiretrovirals are equivalent. The impact of integrase inhibitors on weight gain has been demonstrated, suggesting that pregnant women under treatment with this type of drug have an increased risk of developing metabolic complications, including weight gain and rare events of hyperglycemia, in particular those women who start the pregnancy with a BMI > 25 kg/m^2^. There is little evidence about their progeny; some of them present a rate of newborns that are small for gestational age (SGA) of 15% higher than that expected for the general population, and other publications only infer that the metabolic effects demonstrated in the pregnant woman could also be manifested in the fetus, but this has not been confirmed [22,23,24].

Subcutaneous fat tissue, consisting of adipocytes and vascularized connective tissue [25], plays a pivotal role in immunoregulation, metabolism, and endocrine function [26]. It serves as a reservoir for long-term energy storage and secretes substances such as adipokines that regulate insulin sensitivity and systemic glucose metabolism [27]. The observed association between PI-HAART exposure during pregnancy and the reduction in fetal subcutaneous fat and glucose highlights the negative impact of PI-HAART on the fetus. Consequently, clinicians should be aware of the adverse effects of PI-HAART on fetuses and implement early monitoring and interventions to protect fetal health during the perinatal period. In addition, new research lines should investigate these implications in adulthood, including the risk of metabolic disorders and diabetes, in subjects exposed in utero to antiretroviral therapy.

Changes in neonatal fat tissue content have been linked to enduring metabolic consequences, with poorly developed adipose tissue heightening the likelihood of metabolic syndrome and diabetes in adolescence and adulthood [28]. Hence, we advocate for a thorough medical, nutritional, and metabolic monitoring program for offspring born to HIV-positive mothers receiving PI-HAART. These children face an elevated risk of developing metabolic disorders. While acknowledging the vital role of PI-HAART in curtailing vertical HIV transmission, it is equally imperative to remain vigilant about the potential side effects associated with these medications.

### 4.3. Strengths and Limitations of the Study

This study possesses several strengths. It was carefully designed as a comparative study between two groups, allowing for a focused examination of the impact of PI-HAART on fetal subcutaneous fat. Using ultrasound for measurements, along with a standardized protocol, we minimized variability and enhanced the reliability of our data. Additionally, including a control group and a multivariate analysis strengthened the validity of our findings.

However, there are limitations to our study. The sample size is relatively small, which may impact the generalizability of our results. Furthermore, we only examined a specific set of variables and outcomes, and the complexity of the maternal-fetal interaction during PI-HAART exposure may involve additional factors not explored in this study. Long-term follow-up to assess the clinical implications for children beyond the neonatal period was also beyond the scope of this investigation. Another limitation is that the treatment regimen employed is not widely used globally. While our findings are rooted in historical practice in some countries, they provide valuable insights into the potential effects of antiretroviral therapy on fetal health.

PI-HAART has evolved continuously over the years, but our discoveries open an avenue of new research on the effect of PI-HAART on fetal fat distribution and their long-term implications on fetal programming.

### 4.4. Clinical Interpretation

It is worth noting that, while PI-HAART has shown significant success in reducing vertical HIV transmission, our findings suggest the need to consider the short- and long-term implications of PI-HAART exposure on the fetus. Specifically, it raises the question of how long and broad the impact of PI-HAART exposure during pregnancy on the fetus, newborn, infancy, and adulthood is.

Given that the PI-HAART regimen used in our study is the one employed in Mexico and several countries in Latin America, we believe our findings have significant implications in terms of public health policies. Despite the proven efficacy of PI-HAART in reducing vertical HIV transmission, it is essential to consider a more balanced approach that optimizes both maternal and neonatal health while mitigating the potential risks associated with PI-HAART exposure. Furthermore, policymakers in Mexico and Latin America must take these findings into account and work towards implementing a change in current policies. Actions might include the updating and optimization of the PI-HAART regimens, prioritizing both HIV transmission prevention and the long-term health protection of mothers and newborns. While PI-HAART has been a crucial tool in the fight against HIV, ongoing research and the development of therapies that minimize the potential risks associated with its use, especially during pregnancy, are necessary.

## 5. Conclusions

Our study provides valuable insights into the relationship between PI-HAART, fetal subcutaneous fat tissue, and neonatal glycemia. While the findings are based on a treatment regimen that is not universal, they reflect the reality in Mexico and other Latin American countries. Despite the efficacy of PI-HAART in reducing vertical HIV transmission, our results emphasize the need for a more balanced approach to managing pregnant women with HIV in developing countries and open new venues for research on the impact of intrauterine PI-HAART exposure on energy metabolism and fetal programming.

## Figures and Tables

**Table 1:** Characteristics of the study population.

	Control *n* = 17 Mean (Ranges)	HIV Positive *n* = 17 Mean (Ranges)	*p* Value
Maternal age (year)	33 (27–36)	31 (29–33)	0.407
Gestational age at screening (weeks)	36.3 (36.1–36.4)	36.2 (36.1–36.3)	0.636
Gestational BMI (Kg/m^2^)	27.53 (26.02–29.38)	25.78 (24.30–29.38)	0.221
Maternal cholesterol (mg/dL)	261.05 (234–302)	253.11 (204–290)	0.758
Maternal triglycerides (mg/dL)	221.76 (145–310)	230.82 (167–301)	0.362
Fetal weight by ultrasound (g)	2809 (2743–2967)	2712 (2654–2799)	0.105
Newborn weight (g)	2890 (2780–2990)	2832 (2745–2940)	0.191
Abdominal circumference at birth (cm)	31.5 (30–32)	32.5 (32–33)	0.015
Gestational age at birth (weeks)	38.5 (38.3–39)	38.2 (38.1–38.4)	0.261
Preeclampsia	0	1 (5.88%)	0.310
Fetal growth restriction	0	1 (5.88%)	0.310
Apgar 1 min	8 (8–9)	8 (8–9)	0.379
Apgar 5 min	8 (8–9)	8 (8–9)	0.558
Maternal serum glucose at birth (mg/dL)	85.4 (79.8–86.7)	85.3 (79.2–90)	0.783
Newborn serum glucose (mg/dL)	65.7 (62.1–70.3)	56.1 (52.4–65.3)	0.007
Newborn cholesterol (mg/dL)	152.82 (122–178)	151.47 (110–189)	0.373
Newborn HDL cholesterol (mg/dL)	51.23 (42–60)	51.64 (34–62)	0.129
Newborn LDL cholesterol (mg/dL)	70.58 (63–78)	71.88 (62–79)	0.073
Newborn triglycerides (mg/dL)	135.41 (120–160)	137.94 (119–167)	0.910

BMI: body mass index; HDL: high-density lipoprotein; LDL: low-density lipoprotein.

**Table 2:** Fat area in the three axial planes of the humerus and femur among the study groups.

	Control cm^2^, Median (IQR)	HIV Positive cm^2^, Median (IQR)	*p* Value
HUMERUS			
Proximal third-middle union	6.85 (5.45–7.98)	5.6 (5.19–6.06)	0.047
Middle	6.26 (5.79–7.1)	5.34 (5.08–5.81)	0.018
Distal third-middle	5.92 (5.49–6.67)	5.1 (4.26–5.63)	0.007
FEMUR			
Proximal third-middle union	11.94 (10.94–12.52)	9.22 (8.64–10.09)	0.001
Middle	9.76 (8.19–11.81)	8.22 (7.24–9.73)	0.016
Distal third-middle	9.51 (8.82–10.32)	7.84 (6.83–8.97)	0.005

Differences in Mann–Whitney *U*-tests. IQR: Interquartile range.

**Table 3:** Association between PI-HAART and primary and secondary outcomes.

	Coefficient	95%CI	*p*-Value
HUMERUS			
Proximal third-middle union	0.955	−1.042–2.954	0.348
Middle	−1.690	−4.324–0.944	0.209
Distal third-middle	−1.704	−4.311–0.904	0.200
FEMUR			
Proximal third-middle union	−2.588	−5.506–0.329	0.042
Middle	0.509	−0.234–3.254	0.090
Distal third-middle	0.897	−0.693–2.488	0.269
Fetal weight	−0.302	−0.106–4.624	0.438
Newborn weight	−0.163	−0.044–0.012	0.254
Newborn abdominal circumference	0.456	−0.561–1.472	0.380
Newborn serum glucose	−1.277	−3.055–0.058	0.045
Maternal BMI	1.794	−1.305–4.895	0.257
Maternal serum glucose	0.019	−0.325–0.364	0.911

CI: confidence interval; BMI: body mass index.

## Data Availability

The data presented in this study are available on request from the corresponding author. The data are not publicly available due to privacy.

## Supplementary Materials

The following supporting information can be downloaded at: https://www.mdpi.com/article/10.3390/v16010010/s1. Figure S1: Volumetry assessments on the fetal arm and thigh; Table S1: Demographic characteristics of the entire study population.

## References

[1] Trindade L.N.M., Nogueira L.M.V., Rodrigues I.L.A., Ferreira A.M.R., Corrêa G.M., Andrade N.C.O. (2021). HIV infection in pregnant women and its challenges for the prenatal care. Rev. Bras. Enferm..

[2] Drake A.L., Wagner A., Richardson B., John-Stewart G. (2014). Incident HIV during pregnancy and postpartum and risk of mother-to-child HIV transmission: A systematic review and meta-analysis. PLoS Med..

[3] Johnson L.F., Mutemaringa T., Heekes A., Boulle A. (2020). Effect of HIV Infection and Antiretroviral Treatment on Pregnancy Rates in the Western Cape Province of South Africa. J. Infect. Dis..

[4] Mofenson L.M., Baggaley R.C., Mameletzis I. (2017). Tenofovir disoproxil fumarate safety for women and their infants during pregnancy and breastfeeding. Aids.

[5] Koss C.A., Natureeba P., Kwarisiima D., Ogena M., Clark T.D., Olwoch P., Cohan D., Okiring J., Charlebois E.D., Kamya M.R. (2017). Viral Suppression and Retention in Care up to 5 Years after Initiation of Lifelong ART during Pregnancy (Option B+) in Rural Uganda. J. Acquir. Immune Defic. Syndr..

[6] Debroy P., Lake J.E., Moser C., Olefsky M., Erlandson K.M., Scherzinger A., Stein J.H., Currier J.S., Brown T.T., McComsey G.A. (2021). Antiretroviral Therapy Initiation Is Associated with Decreased Visceral and Subcutaneous Adipose Tissue Density in People Living with Human Immunodeficiency Virus. Clin. Infect. Dis..

[7] Koethe J.R., Lagathu C., Lake J.E., Domingo P., Calmy A., Falutz J., Brown T.T., Capeau J. (2020). HIV and antiretroviral therapy-related fat alterations. Nat. Rev. Dis. Primers.

[8] Tshamala H.K., Aketi L., Tshibassu P.M., Ekila M.B., Mafuta E.M., Kayembe P.K., Aloni M.N., Shiku J.D. (2019). The Lipodystrophy Syndrome in HIV-Infected Children under Antiretroviral Therapy: A First Report from the Central Africa. Int. J. Pediatr..

[9] Lake J.E., Currier J.S. (2013). Metabolic disease in HIV infection. Lancet Infect. Dis..

[10] Martínez E., Garcia-Viejo M.A., Blanch L., Gatell J.M. (2001). Lipodystrophy syndrome in patients with HIV infection: Quality of life issues. Drug Saf..

[11] Hirsch H.H., Battegay M. (2002). Lipodystrophy syndrome by HAART in HIV-infected patients: Manifestation, mechanisms and management. Infection.

[12] Shinar S., Agrawal S., Ryu M., Walmsley S., Serghides L., Yudin M.H., Murphy K.E. (2022). Perinatal outcomes in women living with HIV-1 and receiving antiretroviral therapy-a systematic review and meta-analysis. Acta Obstet. Gynecol. Scand..

[13] Chilaka V.N., Konje J.C. (2021). HIV in pregnancy—An update. Eur. J. Obstet. Gynecol. Reprod. Biol..

[14] Costa J.M., Torres T.S., Coelho L.E., Luz P.M. (2018). Adherence to antiretroviral therapy for HIV/AIDS in Latin America and the Caribbean: Systematic review and meta-analysis. J. Int. AIDS Soc..

[15] Hernanz-Lobo A., Ruiz Saez B., Carrasco García I., Mino-Leon G., Juárez J., Pavía Ruz N., Estripeaut D., Pérez M., Erazo K., Castaneda Villatoro L.G. (2022). New diagnosis of mother-to-child transmission of HIV in 8 Latin-American countries during 2018. BMC Infect. Dis..

[16] México C.S.d.S. Guía de Manejo Antirretroviral de las Personas con VIH. https://www.gob.mx/cms/uploads/attachment/file/712164/Gu_a_TAR_fe_erratas_2022.pdf.

[17] Saag M.S., Gandhi R.T., Hoy J.F., Landovitz R.J., Thompson M.A., Sax P.E., Smith D.M., Benson C.A., Buchbinder S.P., Del Rio C. (2020). Antiretroviral Drugs for Treatment and Prevention of HIV Infection in Adults: 2020 Recommendations of the International Antiviral Society-USA Panel. JAMA.

[18] Mulligan K., Anastos K., Justman J., Freeman R., Wichienkuer P., Robison E., Hessol N.A. (2005). Fat distribution in HIV-infected women in the United States: DEXA substudy in the Women’s Interagency HIV Study. J. Acquir. Immune Defic. Syndr..

[19] Lu W.L., Lee Y.T., Sheu G.T. (2021). Metabolic Syndrome Prevalence and Cardiovascular Risk Assessment in HIV-Positive Men with and without Antiretroviral Therapy. Medicina.

[20] Nsagha D.S., Assob J.C., Njunda A.L., Tanue E.A., Kibu O.D., Ayima C.W., Ngowe M.N. (2015). Risk Factors of Cardiovascular Diseases in HIV/AIDS Patients on HAART. Open AIDS J..

[21] Van Wijk J.P., Cabezas M.C. (2012). Hypertriglyceridemia, Metabolic Syndrome, and Cardiovascular Disease in HIV-Infected Patients: Effects of Antiretroviral Therapy and Adipose Tissue Distribution. Int. J. Vasc. Med..

[22] Han W.M., Kerr S.J., Avihingsanon A., Boettiger D.C. (2022). Weight change with integrase strand transfer inhibitors among virally suppressed Thai people living with HIV. J. Antimicrob. Chemother..

[23] Fuller T., Fragoso da Silveira Gouvêa M.I., Benamor Teixeira M.L., Ferreira Medeiros A., Amorim da Silva P., Medeiros Braga C., Constan Werneck Sant’anna M., de Mattos Salgueiro M., da Silveira Bressan C., Mendes-Silva W. (2023). Real-world experience with weight gain among pregnant women living with HIV who are using integrase inhibitors. HIV Med..

[24] Dontsova V., Mohan H., Blanco C., Jao J., Greene N.D.E., Copp A.J., Zash R., Serghides L. (2023). Metabolic implications and safety of dolutegravir use in pregnancy. Lancet HIV.

[25] Brüggen M.C., Stingl G. (2020). Subcutaneous white adipose tissue: The deepest layer of the cutaneous immune barrier. J. Dtsch. Dermatol. Ges..

[26] Rosen E.D., Spiegelman B.M. (2014). What we talk about when we talk about fat. Cell.

[27] Tran T.T., Yamamoto Y., Gesta S., Kahn C.R. (2008). Beneficial effects of subcutaneous fat transplantation on metabolism. Cell Metab..

[28] Nakano Y. (2020). Adult-Onset Diseases in Low Birth Weight Infants: Association with Adipose Tissue Maldevelopment. J. Atheroscler. Thromb..

