# Supplementary material for: Impact of Protease Inhibitor-Based Highly Active Antiretroviral Therapy on Fetal Subcutaneous Fat Tissue in HIV-Pregnant Women in a Middle-Income Country
Source: Viruses. 2023 Dec 20;16(1):10. doi: 10.3390/v16010010 (PMC10818469; doi:10.3390/v16010010)
Supplement: Supplementary file 1 [file viruses-16-00010-s001.zip › viruses-2745104-SI.pdf]

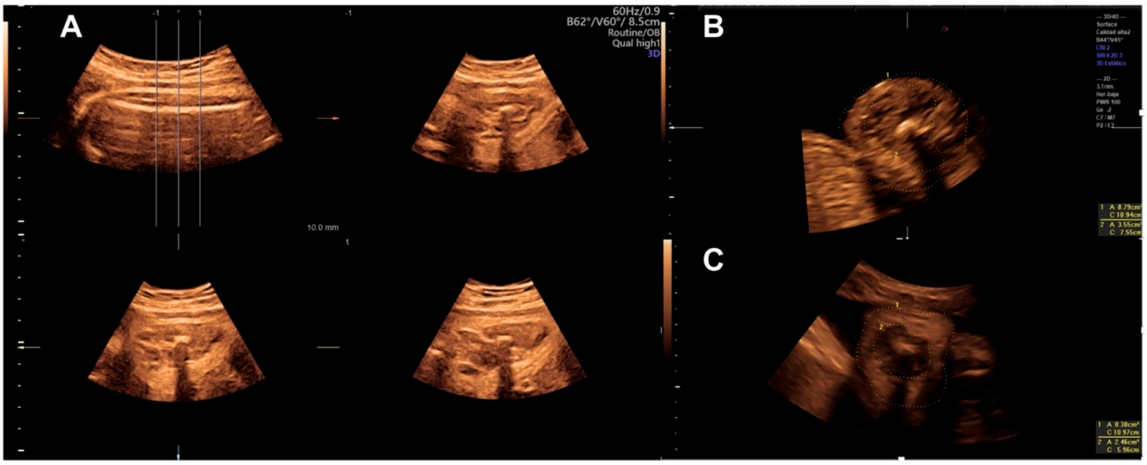

Supplementary Figure S1. Volumetry assessments on the fetal arm and thigh. The transducer was placed as close to the limb as possible without applying pressure **(A)**, ensuring no interference from fetal or maternal movements, we employed a zoom setting to encompass 70 to 80% of the screen area and selected a volume acquisition angle of 80°, ensuring precise centering of the limb. In the subsequent offline assessment phase, we utilized ViewPoint program from GE Healthcare. The sagittal plane of the bone was displayed as the primary screen, with the proximal epiphysis positioned to the left. To enhance the differentiation between lean and fat mass, a Sepia filter was applied. The TUI (Tomographic Ultrasound Imaging) tool was employed to define three tomographic slices, each with the diaphysis centered, including the central position and one to the right and one to the left.

The fat mass area was calculated by subtracting the central area representing lean mass, which includes bone and muscle, from the total area captured in the image. A minimum of two measurements were obtained for each tomographic plane, and the measurement of the highest quality was selected for further analysis. We utilized three distinct planes for the humerus **(B)** and femur **(C)** were utilized: the junction of the proximal third with the middle third, the midpoint of the bone, and the union of the distal third with the middle third.

Supplementary Table S1. Demographic characteristics of the entire study population (n=34)

**Characteristic**	**n**	**%**
**Age** (years) Median (IQR)	31 (27-35)	
**Marital status**		
Married	10	29.4
Single	8	23.5
Live with a partner	16	47.1
**Scholarship**		
High school	11	32.4
Secondary school	17	50
Bachelor´s degrade	3	8.8
Technical career	3	8.8
**Labor**		
Home	29	85.3
Office worker	5	14.7
**Pregnancy number**		
1	16	47.1
2	10	29.4
3 +	8	23.5
**Socioeconomic level**		
Lower-middle class	22	64.7
Middle class	12	35.3
**Gestational age at scan** Median (RIQ).	36.2 (36.1-36.4)	
**Body Mass Index** (kg/m^2) Median (RIQ).	27.15 (24.53-29.38)	
**Gestational age at delivery** Median and (RIQ).	38.3 (38.2-38.6)	
